# Chemical composition of extracts from leaves, stems and roots of wasabi (*Eutrema japonicum*) and their anti-cancer, anti-inflammatory and anti-microbial activities

**DOI:** 10.1038/s41598-023-36402-y

**Published:** 2023-06-05

**Authors:** Katarzyna Dos Santos Szewczyk, Weronika Skowrońska, Aleksandra Kruk, Anna Makuch-Kocka, Anna Bogucka-Kocka, Małgorzata Miazga-Karska, Anna Grzywa-Celińska, Sebastian Granica

**Affiliations:** 1 Department of Pharmaceutical Botany, Medical University of Lublin, Chodźki 1, 20-093 Lublin, Poland; 2 Department of Pharmacognosy and Molecular Basis of Phytotherapy, Medical University of Warsaw, Banacha 1 Street, 02-097 Warsaw, Poland; 3 Department of Pharmacology, Medical University of Lublin, Radziwiłłowska 11, 20-080 Lublin, Poland; 4 Department of Biology and Genetics, Medical University of Lublin, Chodźki 4a, 20-093 Lublin, Poland; 5 Department of Biochemistry and Biotechnology, Medical University of Lublin, Chodźki 1, 20-093 Lublin, Poland; 6 Chair and Department of Pneumonology, Oncology and Allergology, Medical University of Lublin, Jaczewskiego 8, 20-090 Lublin, Poland; 7 Microbiota Lab, Department of Pharmacognosy and Molecular Basis of Phytotherapy, Faculty of Pharmacy Medical, Centre for Preclinical Research, University of Warsaw, Banacha 1 Street, 02-097 Warsaw, Poland

**Keywords:** Cancer, Microbiology, Plant sciences

## Abstract

The purpose of our study was to evaluate the composition of the extracts obtained from the roots and leaves of *Eutrema japonicum* cultivated in Poland. For this purpose, LC-DAD-IT-MS and LC-Q-TOF-MS analyses were used. The results revealed the presence of forty-two constituents comprising glycosinolates, phenylpropanoid glycosides, flavone glycosides, hydroxycinnamic acids, and other compounds. Then, the resultant extracts were subjected to an assessment of the potential cytotoxic effect on human colon adenocarcinoma cells, the effect on the growth of probiotic and intestinal pathogenic strains, as well as their anti-inflammatory activity. It was demonstrated that 60% ethanol extract from the biennial roots (WR2) had the strongest anti-inflammatory, antibacterial, and cytotoxic activities compared to the other samples. Our results suggest that extracts from *E. japonicum* may be considered as a promising compound for the production of health-promoting supplements.

## Introduction

A close connection between nutrition and health has led to a growing interest of researchers in the issue of the significance of foods or food ingredients on specific functions in the body. Functional food, apart from its nourishing characteristics, has also additional attributes such as delaying the ageing process, disease prevention, and optimization of the functioning of sense organs and other systems of the organism. Plants are one of the main components of the human diet and are an important source of components that can be added to other kinds of food in order to enrich their composition. Alternatively, plants alone may be consumed to achieve particular health-promoting effects^1^. Nowadays, plant food, that is considered to be natural, becomes more and more popular for people aiming to live closer to nature. However, many aspects of the effects of the plants used as functionals are still unresolved and many questions about their roles, especially in medicine, remain open. Therefore, further research in this important field is warranted.

Brassicaceae plants are among the most commonly used vegetables worldwide, rich in numerous active ingredients. *E. japonicum* (Miq.) Koidz. (syn. *Wasabia japonica* (Miq.) Matsum., so-called wasabi or Japanese horseradish, belongs to the Brassicaceae family and is one of the family most commonly used as a traditional spice to prevent food poisoning^2^. The taxa is mainly cultivated by either a semiaquatic system or a field one. In nature, it grows on the shaded, wet banks of cold mountain springs and streams^3^. The paste of wasabi rhizomes has been used for a long time in Japanese traditional food such as sushi and sashimi as a pungent spice^4^. Literature studies have shown that besides value as a functional and regular food, extracts from the leaves and roots of *E. japonicum* contains not only allyl isothiocyanate derivatives^5,6^, but also flavonoids^7,8^, phenylpropanoids^8,9^, and carotenoids^10^. In our previous research, we proved that the flowers, leaves, and roots of *E. japonicum* are rich in flavonoids, especially isovitexin 4’-*O*-glucoside, luteolin 3′,7′-diglucoside, and kaempferol 3-*O*-rutinoside^11^. Extracts from the leaves and roots of *E. japonicum* have numerous activities, such as anti-inflammatory^8,10,12^, antigenotoxic^13^, antioxidant^8,9^, anticancer^14,15^, neuroprotective^16^, and anti-obesity properties^17^. Recently, Park and co-authors showed that a new 2-butenolide derivative, isolated from the rots of *W. japonica*, possess strong anti-neuroinflammatory activity by inhibiting NO production in LPS-stimulated BV-2 cells, and α-tocospiro A exhibited a potent antiproliferative activity against A549 non-small cell lung adenocarcinoma cells^18^.

As a response to the increasing requirements for high-quality and safety supplements, there is a growing interest in plant extract products that are rich in many active compounds. In our research, we attempted to demonstrate the benefits arising from the potential use of edible plants, such as *Eutrema japonicum*. For this purpose, different parts of *E. japonicum* cultivated in Poland were investigated. Therefore, the active compounds content was determined, as well as the anti-inflammatory, antimicrobial, and cytotoxic properties.

## Results and discussion

### Qualitative LC-DAD-IT-MS and LC-Q-TOF analysis of chemical composition

**Figure 1:**
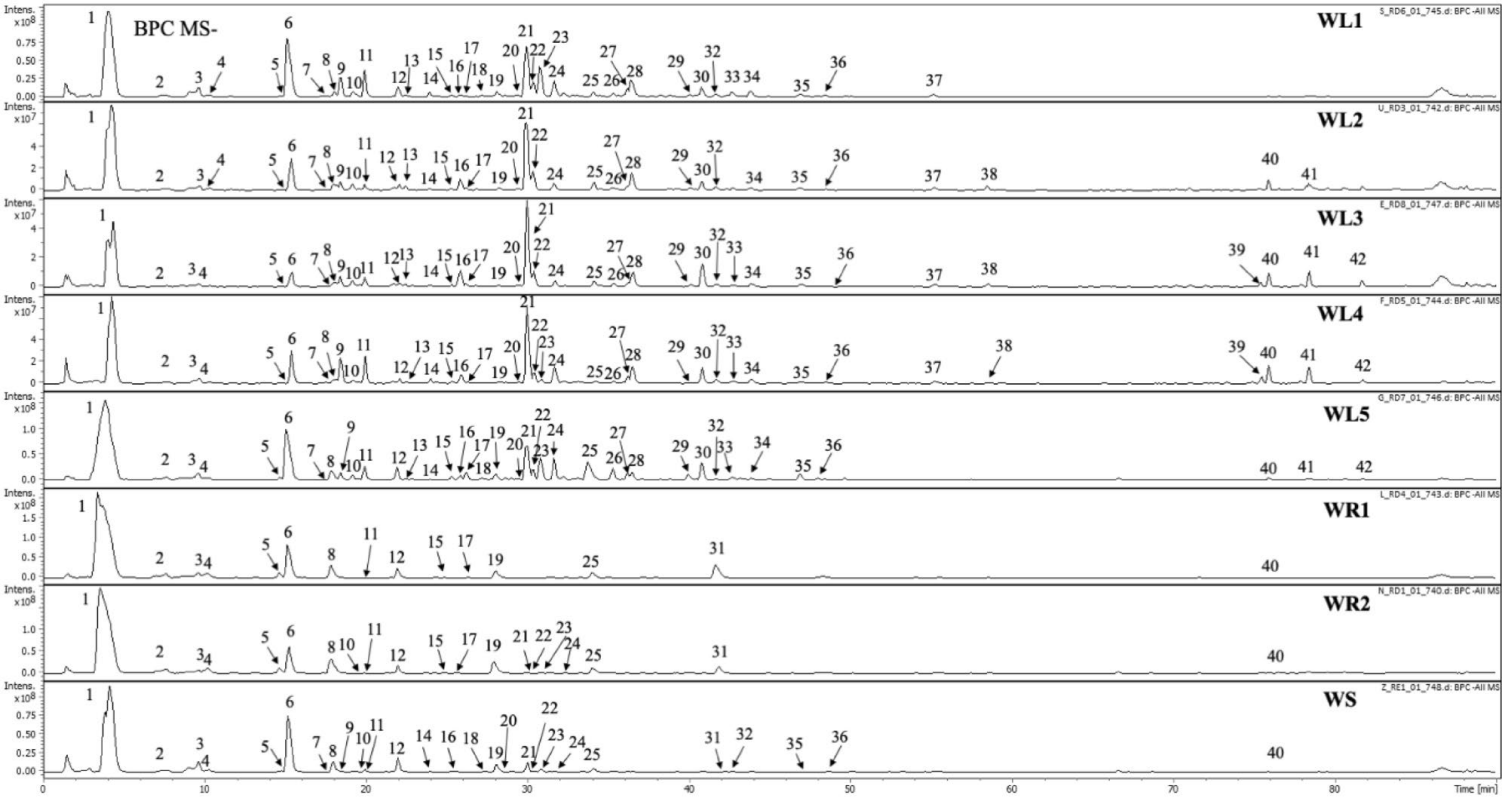
Base peak chromatogram in negative mode of analysed samples obtained in LC-DAD-IT-MS analysis. Methanol-acetone–water extracts from: WL1—annual leaves, WL2—biennial leaves, WS—annual stems, WL5—biennial stems, WR1—biennial roots, and 60% ethanol extracts from: WL4—annual leaves, WL3—biennial leaves, WR2—biennial roots.

The chromatographic analysis extracts from leaves, stems, and roots of *E. japonicum* allowed detection of 42 major peaks observed on BPC MS^-^ chromatograms (Fig. 1, Table 1). Compounds were classified to one of phytochemical groups based on maxima observed in UV–Vis spectra and analysis of fragmentation patterns using ion-trap mass spectrometer. Compounds with no chromophores in the range from 230 to 450 nm were preliminary assigned as glucosinolates (**1**–**6**, **8**, **12**, **16**, **19**, **25** and **31**). Natural products showing UV maxima around 325 nm were preliminary identified as sinapic acid derivatives (phenylpropanoids) including esters with sucrose, gentobiose and other monosaccharides (**7**, **9**, **10**, **13**–**15**, **18**, **23**, **24**, **26**, **27**, **29**, **33**, and **36**). Compounds with intensive absorption maxima around 330–350 nm were classified as flavonoids (**20**–**22**, **28**, **30**, and **35**). Compounds **17** and **37**–**42** could not be assigned to any group or preliminary identified so they were labelled as undefined in Table 1.

**Table 1:** LC-DAD-MS^3^ data for compounds detected in the analysed *E. japonicum* extracts.

	Compound name	Retention time [min]	UV maxima [nm]	[M-H]^-^ m/z	MS^2^ ions	MS^3^ ions	Exact mass [M-H]^-^ m/z	Molecular formula	WL1	WL2	WL3	WL4	WL5	WR1	WR2	WS
1	Sinigrin (2-propenyl glucosinolate)	4.1	226	358	106, 119, 135, 162, 167, 179, 195, 195, 209, 227, 241, 259b, 275	–	358.02684	C_10_H_17_NO_9_S_2_	+++	+++	++	+++	+++	+++	+++	+++
2	1-methylethylglucosinolate	7.7	–	360	119, 145, 168, 182, 195, 198, 227, 241, 259b, 275, 328, 342	–	360.04325	C_10_H_19_NO_9_S_2_	±	±	±	±	±	±	±	±
3	Glucoalyssin (5-methylsulfinylpentyl glucosinolate)	9.7	–	450	135, 162, 208, 259, 275, 291 386b, 388, 435	–	450.05917	C_13_H_25_NO_10_S_3_	**+**	±	±	±	±	±	±	+
4	Gluconapin (3-butenyl glucosinolate)	10.3	–	372	145, 195, 209, 259b, 292, 325, 348	–	372.04412	C_11_H_19_NO_9_S_2_	±	±	±	±	±	±	±	±
5	Glucochlearin (1-methylpropyl glucosinolate)	14.6	213	374	145, 242, 259b, 275, 294	–	374.06128	C_11_H_21_NO_9_S_2_	±	±	±	±	±	±	±	±
6	Glucohesperin (6-methylsulfinylhexylglucosinolate)	15.1	220	464	259, 291, 340b, 401,449,	–	464.07642	C_14_H_27_NO_10_S_3_	+++	++	++	++	+++	++	++	+++
7	Phenylpropanoid glycoside I	17.3	213, 327	533	218, 233, 275, 487,515b	–	533.15298	C_22_H_30_O_15_	±	±	±	±	±	nd	nd	±
8	Glucobrassicanapin (4-pentenyl glucosinolate)	18.1	214, 327	386	163, 208, 227, 259b, 275, 306	–	386.06226	C_12_H_21_NO_9_S_2_	±	±	±	±	±	+	+	+
9	6-caffeoylsucrose	18.5	220, 297, 327	503	161, 179b, 203, 383, 425, 443	–	503.14504	C_21_H_28_O_14_	+	+	+	++	+	nd	nd	±
10	Phenylpropanoid glycoside II	19.2	214, 327	547	427, 457, 487b, 529	–	547.16715	C_23_H_32_O_15_	±	±	±	±	±	nd	±	±
11	1-(3'',4''-dihydroxy-5''-methoxy)-O-trans-cinnamoyl gentiobiose	19.9	217, 329	533	176, 191, 209b, 233, 413, 473	–	533.15148	C_22_H_30_O_15_	++	±	+	++	+	±	±	±
12	Glucoibarin (7-methylsulfinylheptyl glucosinolate)	22.0	213, 318	478	259, 285, 414b	–	478.08623	C_15_H_29_NO_10_S_3_	+	±	±	±	+	+	+	+
13	Phenylpropanoid glycoside III	22.5	214, 322	547	385, 427 457b, 487	–	547.17009	C_23_H_32_O_15_	±	±	±	±	±	nd	nd	nd
14	6-*O*-feruloylsucrose	23.9	214, 330	517	193b, 323	–	517.15615	C_22_H_30_O_14_	±	±	±	±	±	nd	nd	±
15	Phenylpropanoid glycoside IV	25.3	217, 327	547	208, 223b, 277, 323, 488	–	547.17168	C_23_H_32_O_15_	±	±	±	±	±	±	±	nd
16	Glucosinolate I	25.8	217, 336	737	244, 259, 583b	–	737.19728	C_22_H_46_N_2_O_21_S_2_	±	+	+	+	±	nd	nd	±
17	Undefined compound	26.2	217	431	160, 223, 385b	–	431.10345	C_14_H_24_O_15_	±	±	±	±	+	±	±	nd
18	1,2'-di-(3'',4''-dihydroxy-5''-methoxy)-*O*-*trans*-cinnamoyl gentiobiose	27.2	217, 327	725	443, 473b, 515, 533	–	725.19313	C_32_H_38_O_19_	±	nd	nd	nd	±	nd	nd	±
19	5-hexenyl glucosinolate	28.1	217, 331	400	222, 259b, 275, 320, 368	–	400.07632	C_13_H_23_NO_9_S_2_	±	±	±	±	+	+	+	+
20	Isosaponarin with caffeic acid or hexoside	29.4	217, 327	755	293, 412, 455, 593b, 635	293, 335, 413, 473b	755.19413	C_36_H_35_O_18_	±	±	±	±	±	nd	nd	±
21	Isosaponarin ^s^	29.9	195, 213, 271, 326	593	473b, 503	–	593.15463	C_27_H_30_O_15_	+++	+++	+++	+++	++	nd	±	+
22	Undefined flavonoid	30.4	271, 335	609	327, 357, **447b**, 489, 519	429, 411, 387, 371, 357b, 327	609.14580	C_27_H_30_O_16_	+	+	+	±	±	nd	±	±
23	1-(3'',4''-dihydroxy-5''-methoxy)-*O*-*trans*-cinnamoyl-2'-*O*-*trans*-sinapoyl gentiobiose	30.7	197, 206, 223, 324	739	385, 427, 457, 487 515, 529b, 530, 533	–	739.20928	C_33_H_40_O_19_	++	nd	nd	+	++	nd	±	±
24	1-*O*-*trans*-caffeoyl-2'-*O*-*trans*-sinapoyl gentiobiose	31.7	217, 324	709	385, 457, 503, 529b, 530	–	709.20170	C_32_H_38_O_18_	+	+	±	+	++	nd	±	±
25	4-methoxyglucobrassicin	34.1	217, 327	477	140, 195, 203, 259, 275b, 281, 291, 299, 394	–	477.06573	C_17_H_22_N_2_O_10_S_2_	±	+	±	±	++	+	+	±
26	1,2'-di-*O*-*trans*-sinapoyl gentiobiose	35.3	217, 327	753	223, 427, 487, 529b, 530, 693	–	753.22681	C_34_H_42_O_19_	±	±	±	±	+	nd	nd	nd
27	1-*O*-*trans*-feruoyl-2'-*O*-*trans*-sinapoyl gentiobiose	36.2	213, 268, 340	723	487, 529b	–	723.21851	753.22681	±	±	±	±	±	nd	nd	nd
28	Isoorientin^s^	36.4	211, 268, 349	447	326, 327, 357b, 387, 411, 429	–	447.09438	C_21_H_20_O_11_	+	+	+	+	+	nd	nd	nd
29	Sinapinic acid derivative	39.9	217, 321	621	191, 223, 353, 397b, 415, 446	–	621.14447	C_28_H_30_O_16_	±	±	±	±	±	nd	nd	nd
30	Isovitexin^s^	40.8	214, 270, 336	431	311b, 341, 413	–	431.10072	C_21_H_20_O_10_	+	+	++	+	+	nd	nd	nd
31	Neoglucobrassicin (1-methoxy-3-indolylmethyl glucosinolate)	41.6	219, 289	477	144, 291, 383, 446b, 447	–	477. 48,706,540	C_17_H_22_N_2_O_10_S_2_	nd	nd	nd	nd	nd	+	+	±
32	ferulic acid^s^	41.7	217, 324	193	192b, 208	–	193.05210	C_10_H_10_O_4_	±	±	±	±	±	nd	nd	±
33	Phenylpropanoid gycoside V (isomer of compound 23)	42.7	218, 330	739	413, 457, , 503, 529b, 533	–	739.21112	C_33_H_40_O_19_	±	nd	±	±	±	nd	nd	nd
34	sinapinic acid^s^	43.9	221, 323	223	224b, 225	–	223.06438	C_11_H_12_0_5_	±	±	±	±	±	nd	nd	nd
35	7-*O*-*trans*-sinapoylisovitexin-4'-*O*-*β*-D-glucopyranoside	46.9	218, 272, 324	800	413, 431, 593, 594, 637b, 679	–	800.21638	C_38_H_40_O_19_	±	±	±	±	+	nd	nd	±
36	Phenylpropanoid glycoside VI (isomer of compound 27)	48.5	218, 327	723	223, 385, 427, 457, 487, 529b, 663	–	723.20823	C_33_H_40_O_18_	±	±	±	±	±	nd	nd	±
37	Undefined compound	55.2	220, 324	451	181, 241b, 367	–	451.16836	C_16_H_28_N_4_O_11_	±	±	±	±	nd	nd	nd	nd
38	Undefined compound	58.5	218, 330	459	246, 290, 305, 385, 412, 444b, 445	–	459.13317	C_13_H_28_N_6_O_8_S_2_	nd	±	±	±	nd	nd	nd	nd
39	Undefined compound	75.4	222	531	249, 250, 253, 485b,	–	531.28072	C_26_H_44_O_11_	nd	nd	±	±	nd	nd	nd	nd
40	Undefined compound	75.9	221	722	397, 415, 581, 674, 676b	–	722.36831	C_27_H_57_N_5_O_17_	nd	±	±	±	nd	±	±	±
41	Undefined compound	78.4	222	559	253, 277, 513b,	–	559.31361	C_28_H_48_O_11_	nd	±	±	±	±	nd	nd	nd
42	Undefined compound	81.8	222	577	165, 207, 225, 299b, 300	–	577.27381	C_41_H_38_O_3_	nd	±	±	±	±	nd	nd	nd

^s^—comparisons with chemical standard have been made, ^a^ [M-2H]^2−^, ^b^ [M + Na]^+^, *- comparisons with chemical standard have been made, b—base peak (the most abundant ion in recorded spectrum), in bold—ions subjected to MS^3^ fragmentation, “nd”—not detected, “±”—tentatively detected, ‘+’— < 20% based on peak area recorded at 254 nm for all identified, “++”—20–35% based on peak area recorded at 254 nm for all identified “+++”— > 35% based on peak area recorded at 254 nm for all identified.

Compounds **21** with base peak in MS spectrum at m/z = 593 and fragmentation to m/z = 473 and 503 in MS^2^ was preliminary identified as isosaponarin which was previously described in *E. japonicum*^7^. The comparison with the chemical standard confirmed this assignment. Compound **20** with base peak ion at m/z = 755 and intensive fragment in MS/MS at m/z = 593 was tentatively identified as isosaponarin derivative, most probably hexoside or caffeoyl derivative due cleavage of 162 amu fragment. Compounds **28** and **30** with major peaks in MS spectra at m/z = 447 and 431, respectively, and characteristic fragmentation patterns for *C*-glycosidic flavones^19^, were identified as isoorientin and isovitexin. The assignment was confirmed by the comparison of retention times with authentic samples. Compound **35** with base peak ion at m/z = 800 and intensive fragments at 593 and 637 was assigned as 7-*O*-*trans*-sinapoylisovitexin-4'-*O*-*β*-D-glucopyranoside which was previously isolated from *E. japonicum*^7^.

Compounds **32** and **34** with m/z at 193 and 223 and comparison of retention times with chemical standards were identified as ferulic acid and sinapinic acid, respectively.

Compounds **9** and **14** showed major peak in MS at 503 and 517. The fragmentation revealed the cleavage of 324 moiety and production of ions in MS^2^ at 179 and 194. Compound were tentatively assigned as monoesters of sucrose, namely 6-*O*-caffeoylsucrose and 6-*O*-ferulolylsucrose.

Compounds **23**, **24**, **26** and **27** showed intensive signals in the MS spectra at 739, 709, 753 and 723, respectively. The fragmentation patterns were similar for all of them with characteristic signal at m/z = 529 corresponding to monosinapoylgentobiose moiety produced by the cleavage of different *O*-linked fragments. Based on the comparison with previous reports on chemical composition of *E. japonicum* compounds were assigned as 1-(3'',4''-dihydroxy-5''-methoxy)-*O*-*trans*-cinnamoyl-2'-*O*-*trans*-sinapoyl gentiobiose (**23**), 1-*O*-*trans*-caffeoyl-2'-*O*-*trans*-sinapoyl gentiobiose (**24**), 1,2'-di-*O*-*trans*-sinapoyl gentiobiose (**26**) and 1-*O*-*trans*-feruoyl-2'-*O*-*trans*-sinapoyl gentiobiose (**27**)^9^. Compounds **11** and **18** with based peak ion at m/z = 533 or 725 and intensive fragment at 473 were tentatively identified as 1-(3'',4''-dihydroxy-5''-methoxy)-*O*-*trans*-cinnamoyl gentiobiose and 1,2'-di-(3'',4''-dihydroxy-5''-methoxy)-*O*-*trans*-cinnamoyl gentiobiose, respectively according to the previously published data^9^.

**Figure 2:**
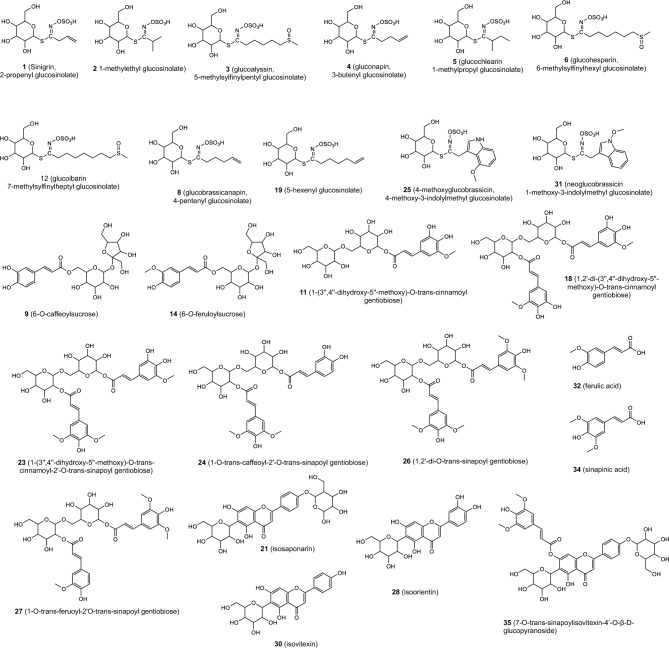
Chemical structures of identified compounds in analysed extracts.

The most diverse group of compounds (**1**–**6**, **8**, **12**, **16**, **19**, **25** and **31)** detected in the analysed extracts were glucosinolates which are typical for *E. japonicum*. The observation of fragmentation patterns as well as eluting order on C-18 column of detected compound in negative ion mode allowed assignment of eleven from them according to the previous literature on the analysis of glucosinolates in other food products^20,21^. Identification of these compounds is given in Table 1. Chemical structures of chemicals detected in the current research is given in Fig. 2.

Among all classes of identified compounds, the compounds occurring in the largest amounts in all studied extracts were sinigrin (2-propenyl glucosinolate) and glucohesperin (6-methylsulfinylhexylglucosinolate). Phenylpropanoid glycoside I and II, 6-caffeoylsucrose, 6-*O*-feruloylsucrose, glucosinolate I, isosaponarin with caffeic acid or hexoside, ferulic acid, 7-*O*-trans-sinapoylisovitexin-4'-*O*-*β*-D-glucopyranoside, and phenylpropanoid glycoside VI were found in all extracts except the roots. Whereas, phenylpropanoid glycoside III, 1,2'-*di-O-trans*-sinapoyl gentiobiose, 1-*O-trans*-feruoyl-2'-*O-trans*-sinapoyl gentiobiose, isoorientin, sinapinic acid derivative, isovitexin, and sinapinic acid were absent in the both extracts from biennial roots and methanol-acetone–water extract from annual stems. 1,2'-di-(3'',4''-dihydroxy-5''-methoxy)-*O-trans*-cinnamoyl gentiobiose was detected only in methanol-acetone–water extracts from annual leaves, annual and biennial stems.

Depending on the extractant used, we obtained different amounts of identified compounds, which is of course justified due to polarity of extractant. It is well known that, the amount of extracted active compounds depends on the polarity of the solvent used, extraction time and temperature, the proportion of plant material to the amount of solvent used, as well as on the qualitative and quantitative composition of the plant material. In ethanol extracts from annual and biennial leaves, as well as from biennial roots, 11 glucosinolates were detected, including, in each of the extracts, large amounts of two of them (sinigrin (2-propenyl glucosinolate, glucohesperin (6-methylsulfinylhexylglucosinolate). Most glucosinolates in the average amount (5, such as glucohesperin (6-methylsulfinylhexylglucosinolate), glucobrassicanapin (4-pentenyl glucosinolate), glucoibarin (7-methylsulfinylheptyl glucosinolate), 5-hexenyl glucosinolate, 4-methoxyglucobrassicin, neoglucobrassicin (1-methoxy-3- indolylmethyl glucosinolate)) was detected in WR2. The highest amount of phenylpropanoids in ethanol extracts was found in WL4 (annual leaves, 13), followed by WL3 (biennial leaves, 12) and WR2 (biennial roots, 4), with high amounts of (6-caffeoylsucrose) occurring only in annual leaves. A similar situation concerned the content of flavonoids in ethanol extracts, where 6 compounds were detected in the extract from one- and two-year-old leaves, and two in the roots. As for the detection of active substances in relation to the part of the plant used, the highest amount of glucosinolates was found in annual stems, phenylpropanoids in annual leaves and biennial stems, and flavonoids in all leaves and biennial stems.

### Cytotoxicity study

**Figure 3:**
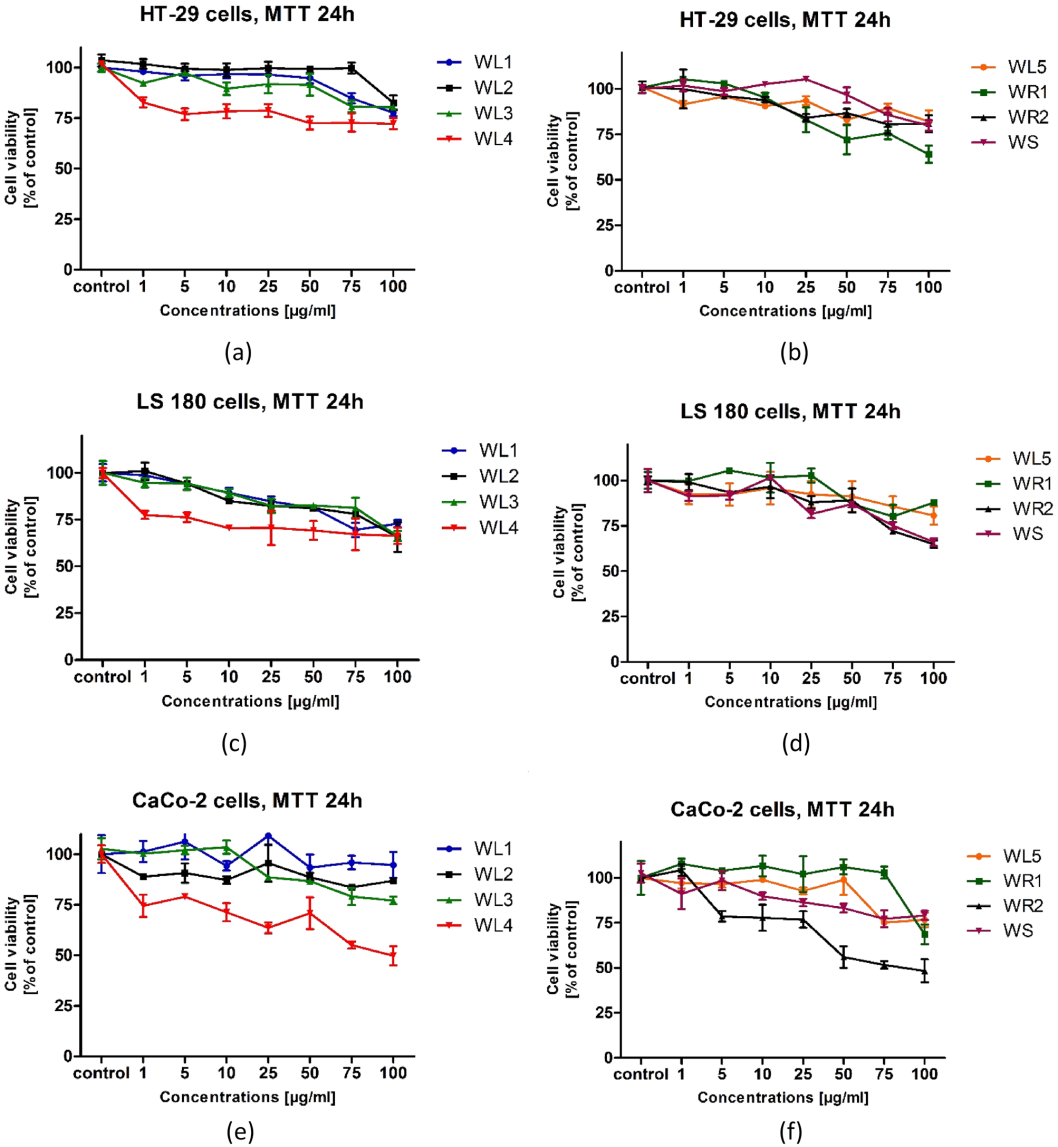
Results of evaluation of cytotoxic effect of samples against chosen cell lines. Results given as mean ± SD. Methanol-acetone–water extracts from: WL1—annual leaves, WL2—biennial leaves, WS—annual stems, WL5—biennial stems, WR1—biennial roots, and 60% ethanol extracts from: WL4—annual leaves, WL3—biennial leaves, WR2—biennial roots.

To test the potential cytotoxic effect of *E. japonicum* extracts on human colon adenocarcinoma cells, HT-29, LS180 and CaCo-2 cells were exposed to serial dilutions of the extracts for 24 h. Our studies did not show high cytotoxic activity of the extracts in the tested concentration range. The viability of all tested cells treated with the extracts was above 50% (Fig. 3a–f). The highest cytotoxicity in relation to the LS-180 and CaCo-2 cell lines was demonstrated by the WR2 extract at a concentration of 100 µg/mL (Fig. 3d,f), while the lowest viability of HT-29 cells was observed when the WR1 extract was added at a concentration of 100 µg/mL to the cells (Fig. 3b).

In previous research it was found that ethanol extract from *Wasabia japonica* and 6-methylsulfinylhexyl isothiocyanate isolated from this extract, induce apoptosis in human monoblastic leukemia U937 cells and human stomach cancer MKN45 cells^22^. Morimitsu et al.^14^ reported that 6-methylsulfinylhexyl isothiocyanate is a good inhibitor of platelet aggregation and has potential anti-cancer activity. This compound inhibits also the development of lung tumors in mice treated with a chemical carcinogen due to the suppression of the initiation stage^23^ and has inhibitory effect on the growth of human stomach tumor cells and on skin carcinogenesis of mice induced by a phorbol ester^15^. Moreover, the results obtained by Hsuan et al.^24^ showed that water extract of *W. japonica* rhizomes has anticancer activity through the induction of apoptosis and autophagy in colon cancer and the induction of the extrinsic pathway and mitochondrial death machinery via the activation of TNF-α, caspases, Fas-L, truncated Bid and cytochrome C.

### Anti-inflammatory activity

COX-1 and COX-2 (cyclooxygenases) are answerable for the transformation of arachidonic acid into several pro-inflammatory mediators, such as prostaglandin H2 (PGH2). Thus, these enzymes play a pivotal role in the inflammatory process^25^.

It has been established that various plants from the Brassicaceae family are capable of inhibiting the activity of COX-1 as well as COX-2 enzyme^26,27^. To the best of our knowledge, just a few reports regarding cyclooxygenase-1 (COX-1) and cyclooxygenase-2 (COX-2) inhibitory activity of *E. japonicum* roots have been conducted^28^. To determine the potential anti-inflammatory activity of *E. japonicum*, we evaluated the ability of the extracts to inhibit the conversion of arachidonic acid to PGH2 by ovine COX-1 and human recombinant COX-2 using a COX inhibitor screening assay kit (Cayman Chemical, MI, USA) (Table 2). The most active extracts against COX-1 were WR2 (IC_50_ = 36.25 ± 0.09 µg/mL) and WR1 (IC_50_ = 40.19 ± 0.15 µg/mL), while the weakest was WS (IC_50_ = 95.17 ± 0.38 µg/mL). In the case of COX-2, WR2 (IC_50_ = 39.70 ± 0.10 µg/mL), WL4 (IC_50_ = 42.18 ± 0.12 µg/mL) and WR1 (IC_50_ = 45.32 ± 0.21 µg/mL) were the most active extracts. However, the activity of tested extracts was lower compared to that of Indomethacin used as a positive control (IC_50_ = 4.51 ± 0.09 μg/mL for COX-1 and 3.95 ± 0.11 μg/mL for COX-2).

**Table 2:** Anti-cyclooxygenase activity of *E. japonicum* extracts.

Sample	IC_50_ [µg/mL]	
	COX-1 inhibition	COX-2 inhibition
WL1	78.50 ± 0.18^a^	61.82 ± 0.21^a^
WL2	64.10 ± 0.20^b^	46.25 ± 0.16^b^
WL3	81.16 ± 0.27^c^	69.10 ± 0.15^c^
WL4	60.29 ± 0.15^d^	42.18 ± 0.12^d^
WL5	52.75 ± 0.18^e^	48.50 ± 0.10^e^
WR1	40.19 ± 0.15^f^	45.32 ± 0.21^f^
WR2	36.25 ± 0.09^g^	39.70 ± 0.10^g^
WS	95.17 ± 0.38^h^	90.53 ± 0.27^h^
IND	4.51 ± 0.09	3.95 ± 0.11

Methanol-acetone–water extracts from: WL1—annual leaves, WL2—biennial leaves, WS—annual stems, WL5—biennial stems, WR1—biennial roots, and 60% ethanol extracts from: WL4—annual leaves, WL3—biennial leaves, WR2—biennial roots. Data given as mean ± SD. Different letters indicate statistically significant differences between means, *p* < 0.05.

### Antimicrobial activity

In addition to its great importance in the processes of digestion, absorption and metabolism, gut microbiota also affects the immune responses of the human body. A lot of research present the connection between various illnesses and intestinal dysbiosis and its mechanisms. Therefore, it is important to keep homeostasis of probiotic strains, then there is less possibility of activation of pathogenic strains of the gastrointestinal tract. The qualitative composition of human and animal food is important in order to provide the probiotic bacteria with a nutrient solution. Such a nutrient medium are prebiotics—mainly plant polysaccharides, but other compounds, including plants rich in glucosinolates, may also have a positive effect on the bacterial homeostase in digestive system.

Isothiocyanates—such as sulforaphane—have strong biological activity in the fight against cancer, as well as cardiovascular and neurodegenerative diseases. They are usually found as glucosinolates in cruciferous vegetables, which themselves are not bioactive until they are degraded by the endogenous enzyme myrosinase to form isothiocyanates. However, most food processing techniques inactivate these myrosinase, so active forms of glucosinolates cannot be produced. Importantly, these active derivatives of glucosinolates can be formed not only by the action of labile plant myrosinases, but there is also an emergency system for their conversion by myrosinase of probiotic bacteria that are part of the gastrointestinal microbiome. Thus, the human microbiome may have a significant impact on increasing the bioavailability of health-promoting glucosinolate derivatives. Tian and co-authors proved that the intestinal microbiota, including *Lactobacillus* spp., act as a mediator to transform glucosinolate precursors in cruciferous vegetables to the active isothiocyanates form^29^.

There are also phytochemicals (forming dietary fibre) that positively modulate the intestinal microbiota. Moreover, many of them (including polyphenols, carotenoids, alkaloids, and glucosinolates) have antioxidant and anti-inflammatory properties^30^.

Importantly, many probiotic strains of the human microbiome use phytochemicals as a source of energy. In addition to treating them as a source of carbon, probiotic strains also use their nitrogen for their growth. Especially the Gram-positive microbiota strains can transfer glucosinolates into beneficial isothyocyanates and nitriles^31^.

On the other hand, the team of Borges et al. proved that glucosinolates have a strong antimicrobial potential against food poisoning bacteria by selective disrupting the cell membranes of these bacteria^32^. Also, Galuppo and co-authors demonstrated the antibacterial activity of glucosinolates against pathogens affecting the health of long-term patients in hospitals^33^.

Thus, it can be concluded that there is a beneficial relationship between glucosinolates and the intestinal microbiome, while inhibiting the growth of pathogenic strains by these phytochemicals and their derivatives.

**Figure 4:**
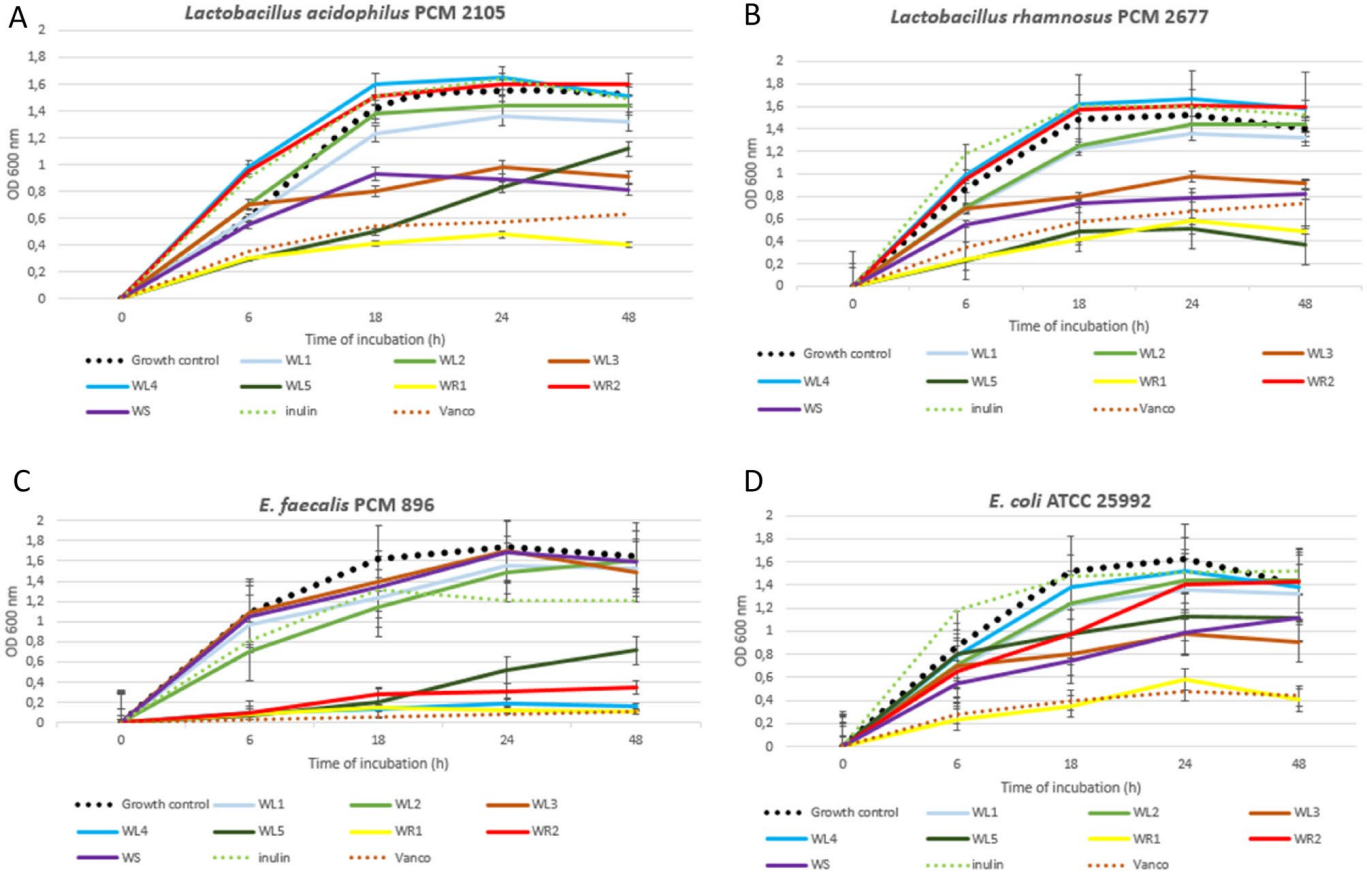
Curves showing the relationships of bacterial growth (OD 600 nm) in the presence of *E. japonicum* extracts or inulin, in comparison with the growth of the test strain, without plant additives. The growth curves for *Lactobacillus acidophilus* PCM 2105 grown in BHI-broth supplemented with *E. japonicum* extracts or inulin. The growth curves were determined by measuring the OD changes at 600 nm for 48 h. (**A**) The growth curves for *Lactobacillus acidophilus* PCM 2105 grown in BHI-broth; (**B**) The growth curves for *Lactobacillus rhamnosus* PCM 2677 grown in BHI-broth; (**C**) The growth curves for *E. faecalis* PCM 896 grown in BHI-broth; (**D**) The growth curves for *E. faecalis* PCM 896 grown in MH-broth.

Extracts WL4 and WR2 can gently activate the growth of beneficial probiotic *Lactobacillus* spp. strains and simultaneously inhibit the growth of strains of *E. faecalis* and *E. coli* that cause gastrointestinal infections. WR1 had a broad antimicrobial activity spectrum because it inhibited the growth of all tested strains, throughout their 48-h growth period studied. The data in Fig. 4A,B,D show that the gold standard of prebiotics (inulin) gently activates the growth of not only probiotic *L. acidophilus* PCM 2105 and *L. rhamnosus* PCM 2677 strains but also pathogenic *E. coli* bacteria. On the other hand, Vancomycin used as an antibiotic standard inhibits the strongest growth of *E. faecalis*, then *E. coli* and then *L. rhamnosus* and *L. acidophilus* strains (Fig. 4A–D). Thomaz et al.^34^ tested the effect of wasabi powder administered to rats on their overall resistance and blood pressure reduction. The authors proved that wasabi supplementation was associated with a significantly increased number of probiotic strains in the rat digestive tract and prevented of arterial hypertension development, they proved therefore, that wasabi powder favourably changes the composition of the intestinal microflora.

## Conclusions

In our in vitro study, we characterized composition and evaluated biological activities of methanol-acetone–water and 60% ethanol extracts from leaves, stems and roots of *E. japonicum*. Thus, we identified the main compounds presenting in extracts as well as we determined cytotoxic, anti-inflammatory, and antimicrobial properties of such extracts. We proved that all extracts are rich in sinigrin (2-propenyl glucosinolate), glucohesperin (6-methylsulfinylhexyl glucosinolate), and isosaponarin, which are most likely responsible for their beneficial biological activities. Among the tested samples, both extracts of biennial roots of *E. japonicum* possessed the most desirable biological properties, as it had the highest cytotoxicity in relation to the human colon adenocarcinoma cells, and antibacterial and anti-inflammatory abilities, while also gently activating the growth of beneficial probiotic *Lactobacillus* spp. strains. A slightly better effect of the ethanol extract from the roots may be due to a slightly different chemical composition compared to the methanol-acetone–water extract. In the ethanol extract, the presence of isosaponarin and one unidentified flavonoid was detected, as well as the phenylpropanoid glycoside II, 1-(3'',4''-dihydroxy-5''-methoxy)-*O*-*trans*-cinnamoyl-2'-*O-trans*-sinapoyl gentiobiose and 1-*O-trans*-caffeoyl-2'-*O-trans*-sinapoyl gentiobiose, which were not observed in the methanol-acetone–water extract. These compounds showed, among others, good radical scavenging activity against superoxide anion radicals (O^•−^_2_) using ESR^9^, significant anti-tumour activity^35,36^, as well as antibacterial^37^ and probiotic activity^38^.

The findings of this study revealed that *E. japonicum* extracts could be used as a readily accessible source of functional food. Furthermore, exploiting such species with anti-inflammatory and antimicrobial capabilities is an ideal goal of future research for the development of food additives and pharmaceutical industry.

## Methods

### Plant material and extraction procedure

Forty grams of leaves (annual and biennial), stems (annual and biennial), and biennial roots of *Eutrema japonicum* (Miq.) Koidz. were collected from the Wasabi Farm Poland in Jedlnia Letnisko (Poland) at an altitude of 160.3 m a.m.s.l., coordinates 51°43′52′′ N, 21°32′27′′ E, in January 2020. Taxonomical identification was confirmed by Prof. K. Dos Santos Szewczyk, the botanist from the Department of Pharmaceutical Botany (Medical University in Lublin, Poland). Voucher specimen was deposited in the Department of Pharmaceutical Botany (EJ-140120). The collection of the plant material and related research complies with relevant institutional, national and international guidelines and legislation.

The plant materials were dried in the shade at 22 °C (± 0.5 °C) to achieve a constant weight. Extracts were prepared using a mixture of methanol–acetone–water (3:1:1, v/v/v; 3 × 50 mL) or 60% ethanol, and were then sonicated at a controlled temperature (40 ± 2 °C) for 30 min. The combined extracts were filtered, concentrated under reduced pressure, and, after freezing, lyophilized in a vacuum concentrator (Free Zone 1 apparatus; Labconco, Kansas City, MO, USA) to obtain dried residues. Dry extracts were weighted and stored in a freezer at − 20 °C. The following yields were obtained: methanol-acetone–water extracts from: annual leaves (WL1)—5.52 g, biennial leaves (WL2)—5.81 g, annual stems (WS)—4.94 g, biennial stems (WL5)—5.36 g, and biennial roots (WR1)—9.67 g, and 60% ethanol extracts from: annual leaves (WL4)—4.27 g, biennial leaves (WL3)—4.65 g, and biennial roots (WR2)—6.77 g.

### Cell cultures and MTT assay

LS180 and HT-29 cell lines were ordered from the European Collection of Cell Cultures (ECACC, Salisbury, UK) and CaCo-2 cell line was ordered from the American Type Culture Collection (ATCC, Menassas, VA, USA). 1:1 mixture of Dulbecco’s Modified Eagle Medium (DMEM) and Ham F-12 nutrient mixture supplemented with 10% Fetal Bovine Serum (FBS) was used to culture LS-180 cells and HT-29 cells. CaCo-2 cell line was cultured using Eagle's Minimal Essential Medium (EMEM) with 20% fetal bovine serum. To culture medium added 100 U/mL of penicillin, and 100 µg/mL of streptomycin. Cell lines were cultured under standard conditions (37 °C, 5% CO_2_).

Stock solutions of the examined extracts were prepared by dissolving the appropriate amount of the extract in sterile dimethylsulfoxide (DMSO). Suspensions of CaCo-2, HT-29 and LS180 cells (a density of 5 × 10^4^ cells/mL) were transferred to 96-well cell culture plates (NUNC, Roskilde, Danmark). After 24 h, the culture medium was removed and either fresh medium with the appropriate concentration of extracts (1, 5, 10, 25, 50, 75, 100 µg/mL) or medium without any treatment (control) was added. Cells were incubated for 24 h under standard conditions. DMSO at the concentrations present in the appropriate dilutions of the stock solutions was not cytotoxic for cell lines. After 24 h of incubation, 15 μL MTT working solution (5 mg/mL in PBS) was added to each well. After 3 h, 100 μL of a SDS buffer (10% SDS in 0.01 N HCl) was added to each well. Cells with MTT and SDS were incubated in 37 °C. The absorbance was measured after 24 h at 570 nm using a microplate reader (Epoch, BioTek Instruments, Inc., USA). Gen5 software (v. 2.01, BioTek Instruments, Inc.) was used. The data were expressed as the percentage of the control.

### Cyclooxygenase-1 (COX-1) and Cyclooxygenase-2 (COX-2) inhibitory activity

The extracts of *E. japonicum* were examined for cyclooxygenase-1 (COX-1) and cyclooxygenase-2 (COX-2) inhibitory activity using a COX (ovine/human) Inhibitor Screening Assay Kit (Cayman Chemical, MI, USA) according to the protocol of the manufacturer. The extracts were tested at different concentrations (20–100 µg/mL). Indomethacin (1 µg/mL) was used as a positive control.

### Assessment of the effect of *E. japonicum* extracts in the bacterial medium on the growth of probiotic strains and intestinal pathogenic strains

*Lactobacillus acidophilus* PCM 2105, *Lactobacillus rhamnosus* PCM 2677 (as probiotic strains) and *Enteroccocus faecalis* PCM 896 and *Escherichia coli* ATCC 25,992 (as enteric pathogenic strains) were used in the experiment. *E. coli* strains were incubated aerobically in Muellera-Hinton-broth (MH-broth or MH-agar) at 37 °C for 48 h. *E. faecalis* and *Lactobacillus* spp. were grown on Brain Heart Infusion broth (BHI-broth or BHI-agar) (Biomaxima, Poland) under the conditions of microaerobic at 37 °C for 48 h. AnaeroGenTM Compact Thermo scientific (Oxoid England) atmospheric generators were used to create incubation under unaerobic conditions.

The assay was performed in a 96-well plate, based on available method^39,40^ with some modifications. Namely 200 μL of the appropriate medium was poured into each well and separately *E. japonicum* extracts (100 µg/mL) were added—to a final concentration of 1.5%—as potential prebiotics. Next, 2 μL of bacterial inoculum (0.5 McFarland, CFU 1.5 × 10^8^) were added to each well and plates were incubated under the optimal conditions, as outlined above.

In the experiment, 1.5% inulin (Sigma-Aldrich, USA) as standard prebiotic and 1.5% Vancomycin hydrochloride (Sigma-Aldrich, USA) as an antibiotic standard were used. As a positive control, a bacterial inoculum in appropriate broth was prepared without any additional extracts. The blank controls were the broth with coloured *E. japonicum* extracts. The broth was used as the negative control. The growth changes of each strain were monitored after 6 h, 18 h, 24 h and 48 h by measuring the optical density (OD) of the cultures at 600 nm, using microplate reader Bio Tech Synergy (USA) with a proprietary software system.

### LC-DAD-IT-MS and LC-Q-TOF–MS analysis

The LC-DAD-IT-MS analysis was performed using Ultimate 3000 series system (Dionex, Idstein, Germany) equipped with diode array detector and coupled with Amazon SL ion trap mass spectrometer (Bruker Daltonik GmbH, Bremen, Germany). The separation of compounds in analyzed samples was achieved on Kinetex XB-C_18_ column (150 mm × 2.1 mm × 1.9 mm), Phenomenex (Torrance, CA, USA). Column temperature was maintained at 25 °C. Elution was conducted using mobile phase A (0.1% formic acid in deionized water) and mobile phase B (0.1% formic acid in acetonitrile) with a multi-step gradient as follows: 0 min 1% B, 60 min 26% B, and finally 90 min 95% B. The flow rate was set to 0.300 mL/min. Three ml of each sample was injected. UV–Vis’s spectra were recorded in the range of 200–450 nm to establish maxima for detected compounds. The eluate was introduced into mass spectrometer without splitting. The ion trap Amazon SL mass spectrometer was equipped with ESI interface. The parameters for ESI source were—nebulizer pressure 40 psi; dry gas flow 9 L/min; dry temperature 300 °C; and capillary voltage 4.5 kV. Compounds were analyzed in negative ion mode only. The MS^2^ and MS^3^ fragmentations were performed using Smart Frag mode. The LC-Q-TOF analysis was performed with Agilent 1260 Infinity apparatus coupled with 6530B Accurate-mass-QTOF-MS (Agilent, equipped with ESI Agilent Dual Jet Stream with the following settings—fragmentor 140 V, capillary voltage 4000 V, gas temperature 300 °C/325 °C and gas flow 12 L/min. Separation condition were same as during LC-DAD-IT-MS run. Samples were dissolved in methanol:water (1:1, v/v) to obtain 10 mg/mL of analyzed extract. Two μl of tested sample was injected to HPLC column.

### Statistical analysis

All measurements were performed at least in triplicate and expressed as means ± standard deviations (± S.D.). Statistical significance was estimated through HSD Tukey’s test for the data obtained from three independent samples of each extract in three parallel experiments (n = 9). Besides the classical pairwise correlation check, we applied the scaled principal component analysis.). Statistical tests were performed using Statistica 6.0 software (StatSoft, Inc., Tulsa, USA).

## Data Availability

The datasets generated or analysed during this study available from the corresponding author on reasonable request.
